# Morphogenesis signaling components influence cell cycle regulation by cyclin dependent kinase

**DOI:** 10.1186/1747-1028-4-12

**Published:** 2009-07-01

**Authors:** Brian TD Tobe, Ana A Kitazono, Jacqueline S Garcia, Renee A Gerber, Brooke J Bevis, John S Choy, Daniel Chasman, Stephen J Kron

**Affiliations:** 1 Ludwig Center for Metastasis Research, University of Chicago, Chicago, IL 60637, USA; 2 Department of Psychiatry, University of California-San Diego, San Diego, CA 92093, USA; 3 Department of Biochemistry and Cellular and Molecular Biology, University of Tennessee, Knoxville, TN 37996, USA; 4 Brigham and Women's Hospital, 900 Commonwealth Avenue, Boston, MA 02115, USA

## Abstract

**Background:**

The yeast cell cycle is largely controlled by the cyclin-dependent kinase (CDK) Cdc28. Recent evidence suggests that both CDK complex stability as well as function during mitosis is determined by precise regulation of Swe1, a CDK inhibitory kinase and cyclin binding partner. A model of mitotic progression has been provided by study of filamentous yeast. When facing nutrient-limited conditions, Ras2-mediated PKA and MAPK signaling cascades induce a switch from round to filamentous morphology resulting in delayed mitotic progression.

**Results:**

To delineate how the dimorphic switch contributes to cell cycle regulation, temperature sensitive *cdc28 *mutants exhibiting constitutive filamentation were subjected to epistasis analyses with *RAS2 *signaling effectors. It was found that Swe1-mediated inhibitory tyrosine phosphorylation of Cdc28 during filamentous growth is in part mediated by Ras2 activation of PKA, but not Kss1-MAPK, signaling. This pathway is further influenced by Cks1, a conserved CDK-binding partner of elusive function with multiple proposed roles in CDK activation, transcriptional regulation and ubiquitin-mediated proteasome degradation.

**Conclusion:**

The dynamic balance between Cks1- and Swe1-dependent regulation of Cdc28 and, thereby, the timing of mitosis during yeast dimorphism is regulated in part by Ras2/cAMP-mediated PKA signaling, a key pathway controlling filamentous growth.

## Background

Yeast cell division is tightly coupled to morphology. Progression through mitosis activates an apical-isotropic switch in which polarized bud growth is redirected to produce round daughter cells [1]. Conversely, mitotic delay results in filamentous growth allowing spreading away from the colony center. Thus, filamentation may be elicited by mutations in mitotic regulators such as the cyclin-dependent kinase (CDK) Cdc28 or its mitotic cyclin binding partner Clb2 [2-4]. Cdc28/Clb2 complexes phosphorylate hundreds of effectors containing S/TP sites, preferably (S/T)PX(K/R), including mitotic spindle components, signaling proteins and other cell cycle regulators [5,6].

The switch from round to filamentous growth is stimulated physiologically by environmental conditions including low nitrogen availability or fusel alcohol exposure [7]. Although it has been hypothesized that these stimuli impinge on CDK and/or its regulators, no clear mechanism has emerged. Extracellular signals are conveyed through a member of the highly conserved RAS GTPase family, Ras2. Deletion of *RAS2 *diminishes filamentation, while the dominant allele *RAS2-VAL19 *induces constitutive pseudohyphal differentiation [2]. Ras2 activates both the Sterile (Ste) Mitogen-Activated Protein Kinase (MAPK) and the Protein Kinase A (PKA) – cAMP cascades [8]. The MAPK pathway involves sequential activation of the PAK kinase Ste20, the MAPKKK Ste11, the MAPKK Ste7 and the MAPK Kss1, culminating in the activation of the transcription factors Tec1 and Ste12 [9,10]. Accordingly, overproduction of Tec1 induces filamentation [11], while deletion of *TEC1 *decreases filament formation [12]. On the other hand, in the PKA pathway, the PKA subunit Tpk2 specifically activates filamentation by activation of the transcription factor Flo8 and inhibition of the transcriptional repressor Sfl1 [13,14]. Deletion of *FLO8 *diminishes filamentation, whereas deletion of *SFL1 *enhances filamentation [12]. Production of cAMP is opposed by phosphodiesterases Pde1 and Pde2 which when overexpressed reverse some *RAS2-V19 *phenotypes [15,16].

One possible target of Ras2 signaling may be the Wee kinase Swe1. Swe1 inhibits Cdc28-Clb2 activity at G2/M via phosphorylation of Cdc28 Tyr19 and a phosphorylation-independent mechanism [17]. Overexpression of Swe1 or stabilization of Swe1 delays mitosis and promotes cell elongation and hyperfilamentation [17-19]. Tyrosine-19 may make direct contact with ATP [20], and it is thought that phosphorylation and even binding by Swe1 impedes ATP utilization by Cdc28. In turn, initial Swe1 phosphorylation by Cdc28/Clb2 complexes stabilizes its interaction with Cdc28, potentiating further inhibition. Inhibition is alleviated by Mih1, a CDK tyrosine phosphatase, which reactivates Cdc28 to hyperphosphorylate Swe1 leading to its dissociation from CDK complexes [21]. Swe1 is also controlled by the Nim1 kinase Hsl1. Deletion of *HSL1 *results in increased Cdc28 tyrosine-19 phosphorylation [22] and enhanced filamentation [17,18], whereas overexpression of *HSL1 *leads to diminished Swe1 protein levels [23]. Further, deletion of *HSL1 *restores filamentation in the absence of *FLO8 *and to a lesser degree *TEC1*, but not in *flo8 tec1 *double mutants [18]. This suggests that PKA has greater specificity for Swe1 inhibition of Cdc28.

PKA has also been shown to mediate Ras2 and glucose-dependent inhibition of the anaphase-promoting complex (APC) [24,25]. The APC is a ubiquitin-protein ligase complex that functions to target proteins for destruction by the proteasome [26]. Overexpression of both the APC-activator *CDC20 *as well as negative regulators of the PKA pathway *PDE2 *and *BCY1 *were found to suppress the temperature sensitivity of a cohesin mutant [27]. Further analysis showed that inhibition of PKA also suppressed temperature sensitivity of *cdc16-1 *mutant defective in APC function [27]. Together these studies indicate the possibility that PKA may negatively regulate the APC. Such a mechanism may further separate the role of PKA from Kss1-MAPK in the regulation of filamentous growth.

Importantly, Cdc28 is also intimately linked to APC activation. Cdc20-dependent APC activity during mitosis is positively regulated via phosphorylation by Cdc28 [28]. Reduced binding of Cdc20 to the APC has been observed in the presence of *cdc28-Y18V, T19F*, a mutant resistant to Swe1 mediated phosphorylation [28]. Further, the CDK subunit Cks1 is required for transcription of *CDC20*. The precise mechanism remains obscure but has been proposed to involve Cks1 recruitment of the proteasome to Cdc28 complexes [29]. Cks1 has been shown to physically interact with proteasome subunits [30]. Interestingly, while certain *cks1 *temperature-sensitive alleles confer G2/M delay [31], Clb cyclin-Cdc28 complexes do not require Cks1 for normal kinase activity [31,32].

To further elucidate the pathway by which extracellular signals impinge on cell cycle, we screened for temperature sensitive *cdc28 *mutants that perform enhanced filamentous growth. A preponderance of the mutations localized to the binding region of Cks1. We examined the genetic interactions of these mutants with cell cycle regulators and Ras2 signaling components. Our results indicate that the PKA-cAMP pathway may be an additional signaling mechanism by which Swe1 inhibition of Cdc28 is affected. However, Swe1 function is downregulated by a mechanism requiring association of Cdc28 with Cks1.

## Results

### cdc28 mutants suggest Cks1 is a regulator of filamentation

We hypothesized that constitutively filamentous *cdc28 *mutants, which experience growth arrest at elevated temperatures would be valuable tools to identify connections between cell cycle and extracellular signals. Therefore, a *cdc28 *shuffle strain was created in which homozygous replacement of *CDC28 *with *TRP1 *gene was covered by a *URA3 *marked plasmid harboring *CDC28*. Transformation of this strain with a library of degenerate-PCR derived *cdc28 *alleles on a *HIS*3 marked plasmid, followed by negative selection against *URA3 *allowed screening colonies for mutants exhibiting enhanced filamentous growth on low nitrogen media. Five hundred colonies were selected [3]. Secondary screening of these colonies for those that exhibited temperature sensitive growth at 35°C revealed 39 mutants with one to five amino acid substitutions.

**Figure 1:**
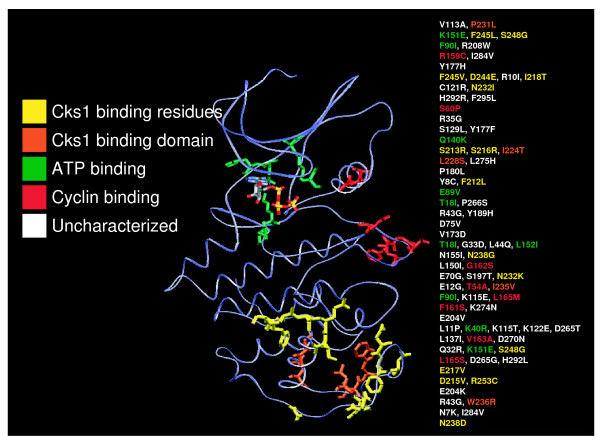
**Hyper-filamentous, temperature sensitive *cdc28 *mutants exhibit altered Cks1-binding region**. The three-dimensional structure of Cdc28 was modeled from the crystal structure of human CDK2 [3]. Each allele is listed with amino acid substitutions color coded according to the functional region of the molecule in which it lies: yellow = Cks1 binding, orange = non-surface residues underlying the Cks1-binding domain, green = ATP-binding, red = cyclin-binding, white = uncharacterized structure function relationship, striped = ATP.

Cyclin-dependent kinase is a bi-lobed protein with an amino terminal domain composed primarily of β-sheets and a larger carboxy-terminal domain containing several α-helices. Between these two domains lies the catalytic core, the opening of which is covered by the T-loop (activation loop) in the inactive monomeric state [20,33-35]. We have previously modeled Cdc28 based on the crystal structure of the human CDK2 enzyme [3,36]. Mapping the residues modified in the selected mutants on our model revealed that while some fell within the interior of the molecule, many were surface residues predicted to participate in binding to known essential partners (Fig. 1). Several mutations fell close to or within the ATP binding cleft (Fig. 1, green) and thus near Tyr19, the target of Swe1 mediated phosphorylation. Other mutations fell in the PSTAIRE helix involved in cyclin binding and catalytic function, or the activation loop, a domain involved in substrate recognition that includes Thr169, the target of Cak1 phosphorylation (Fig. 1, red). However, nearly one third of the alleles contained mutations that might impinge on Cdc28 interaction with Cks1 by altering contact residues, or residues underlying the Cks1 binding surface (Fig. 1, yellow and orange).

We were struck by the high number of mutations potentially affecting Cks1 binding, as this factor had yet to be directly linked to the apical-isotropic switch or filamentous differentiation. We examined if normal growth could be restored in these mutants by high copy *CKS1*. Indeed, 11 out of 15 alleles exhibited improved growth by *CKS1 *overexpression (Table 1). Of the 11 mutants suppressed by *CKS1*, four exhibited near wild type restoration of growth and morphology (1). The point mutants *cdc28-E217V *and *cdc28-N238D *were in this category suggesting that these alterations were less likely to directly affect additional partner associations. Although both mutations lie in the Cks1 binding region, co-crystal studies of the CDK2-Cks1 interface have demonstrated a direct contact only for the residue analogous to Cdc28 Glu217 with residues Glu63 and His94 in Cks1 [37]. Importantly, our bioinformatic analysis using algorithms designed by Chasman and Adams for assessment of the functional consequences of amino acid substitutions [38], indicated that mutation of residue E217 to valine should not otherwise affect the structure of monomeric Cdc28 (data not shown). E217 falls in the highly conserved GDS**E**ID (214–219) motif, previously implicated in Cdc28 binding to Cks1 [39]. Mutations in residues 212, 213, and 215–218 were also identified by this screen, indicating that this domain of Cdc28 is particularly important for regulating morphological differentiation (Fig. 1).

**Table 1:** Genetic comparison of temperature sensitive alleles containing mutations in the Cks1 binding domain

**Cks1 Binding Domain Mutations**	**Suppression by *CKS1 *overexpression**	**Suppression by *flo8***
V113A, P231L	+	-

K151E, F245L, S248G	+	-

F245V, D244E, R10I, I218T	++	-

C121R, N232I	-	-

S213R, S216R, I224T	-	-

L228S, L275H	+	-

Y8C, F212L	+	+

N155I, N238G	+	+

E70G, S197T, N232K	++	+

E12G, T54A, I235V	-	+

Q32R, K151E, S248G	-	-

E217V	++	+

D215V, R253C	+	+

R43G, W236R	+	-

N238D	++	+

*HIS3*-marked plasmids (pRS413) containing temperature sensitive *cdc28* alleles were introduced into a *CDC28* shuffle strain. Transformants were subjected to 5-FOA treatment to evict the wild type copy of *CDC28* and assayed for growth at 25°C and 35°C. If growth of a given allele was significantly improved by deletion of *FLO8* or by overexpression of *CKS1*, then suppression is indicated by "+". A sign of "-" indicates no change.

**Figure 2:**
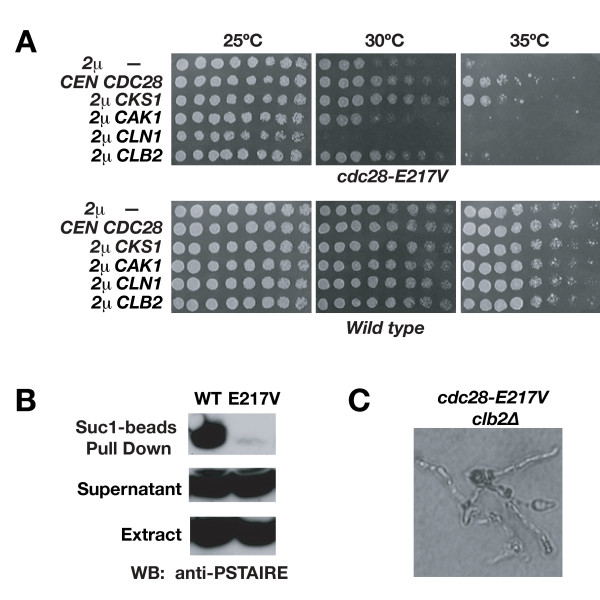
***cdc28-E217V *cells exhibit specific genetic interactions with cell-cycle regulators**. **A) **Growth of *cdc28 *mutant yeast cells containing genomic substitution of E217V transformed with high-copy plasmids harboring *CKS1*, *CAK1*, *CLN1*, *CLB2 *or no insert, or a centromeric plasmid harboring *CDC28*. **B) **p13^Suc1 ^beads-pull downs using yeast protein extract prepared from wild-type or *cdc28-E217V *at 30°C. Blots were probed with anti-PSTAIRE CDK antibody. **C) **Inviability and elongated morphology of *clb2 cdc28-E217V *double mutant segregants.

To better study the genetic interactions of *cdc28-E217V*, we performed genomic replacement of endogenous *CDC28 *with *cdc28-E217V*. As expected, transformation of *cdc28-E217V *with high copy *CKS1 *similarly relieved mutant phenotypes as previously found in the *cdc28-E217V *shuffle strain (Fig. 2A). We also observed decreased pull-down of Cdc28-E217V compared to wild-type Cdc28 with beads coupled to the *S. pombe *Cks1 homolog p13^Suc1^, under conditions of saturating amounts of Cdc28 and Cdc28-E217V proteins (Fig. 2B). These data strongly suggest a loss of association of Cdc28 with its essential partner Cks1 as the basis for the temperature sensitivity in this mutant. Cdc28-E217V also showed normal Cdc28 protein abundance and levels of Clb2- associated histone H1 kinase activity at permissive and restrictive temperatures (data not shown), in accordance with previous reports indicating that Cks1 does not affect mitotic CDK kinase activity [32].

However, we further questioned if the defects of this mutant were specifically attributable to its diminished interaction with Cks1. As a means to examine this possibility we tested overexpression of the CDK-activating kinase *CAK1 *in *cdc28-E217V *cells. Cak1 is an essential CDK activator that phosphorylates Cdc28 on Thr169 in the T loop to promote kinase activity, substrate accessibility and cyclin binding [40,41]. If Cak1 overexpression were to alleviate *cdc28-E217V *defects, it could indicate that *cdc28-E217V *harbors intrinsic defects in CDK function or in interaction with other binding partners or substrates. However, high copy *CAK1 *neither suppressed nor enhanced *cdc28-E217V *defects (Fig. 2A). Dephosphorylation of Thr169 has been shown to be likely mediated by phosphatases Ptc2 and Ptc3 [42]. We found that overexpression of *PTC2 *and *PTC3 *was similarly toxic to wild-type and *cdc28-E217V *cells (data not shown). Thus, *cdc28-E217V *phenotypes are not affected by alteration of activating phosphorylation of Cdc28. This further substantiates that *cdc28-E217V *defects are specific to interaction with Cks1.

Given the well-established roles of Cln1 and Clb2 cyclins at the G1/S and the G2/M transitions respectively, as well as their opposing effects on the apical-isotropic switch, we examined modulation of *cdc28-E217V *phenotypes by *CLB2 *and *CLN1*. *CLB2 *overexpression partly restored *cdc28-E217V *growth at semi-permissive temperature (Fig. 2A) while *cdc28-E217V clb2* double mutant segregants were inviable, germinating to form only elongated, terminal buds (Fig. 2C). In turn, *CLN1 *overexpression diminished proliferation at semi-permissive temperature (Fig. 2A) while deletion of *CLN1 *partly restored growth (data not shown). These results further support that the phenotype of *cdc28-E217V *is due to delayed mitotic progression.

### Protein Kinase A pathway impinges on Cdc28

**Figure 3:**
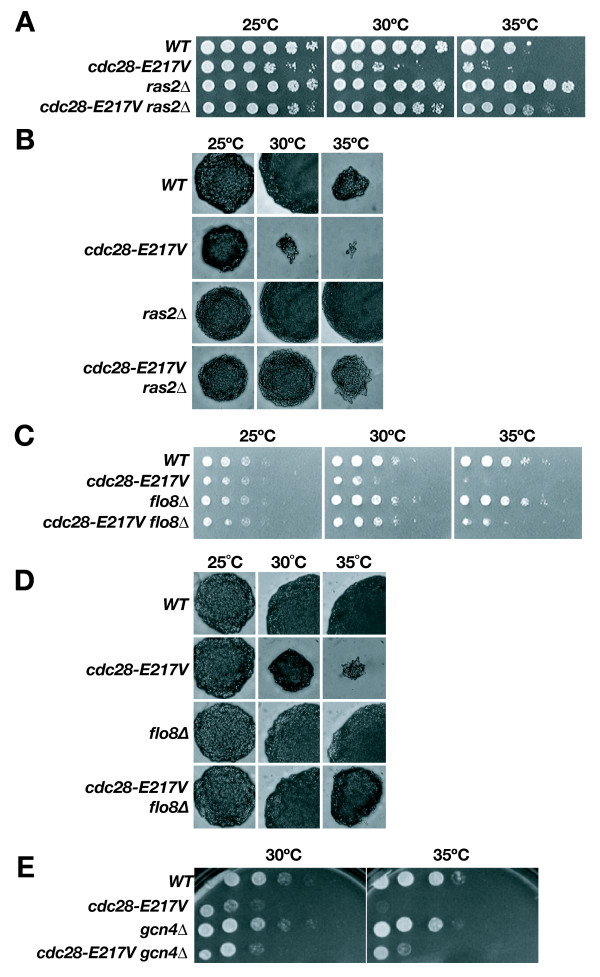
**Ras2-mediated PKA pathway impinges on Cdc28**. **A) **Growth and **B) **Morphology of *cdc28-E217V ras2*, wild-type, *cdc28-E217V*, or *ras2 *cells growing at 25, 30 or 35°C. **C) **Growth and **D) **morphology of *cdc28-E217V flo8 *double mutants compared to wild-type, *cdc28-E217V *and *flo8 *cells at 25, 30 or 35°C. **E) **Growth of *cdc28-E217V gcn4 *double mutants compared to wild-type, *cdc28-E217V *and *gcn4 *cells at 30°C and 35°C.

**Figure 4:**
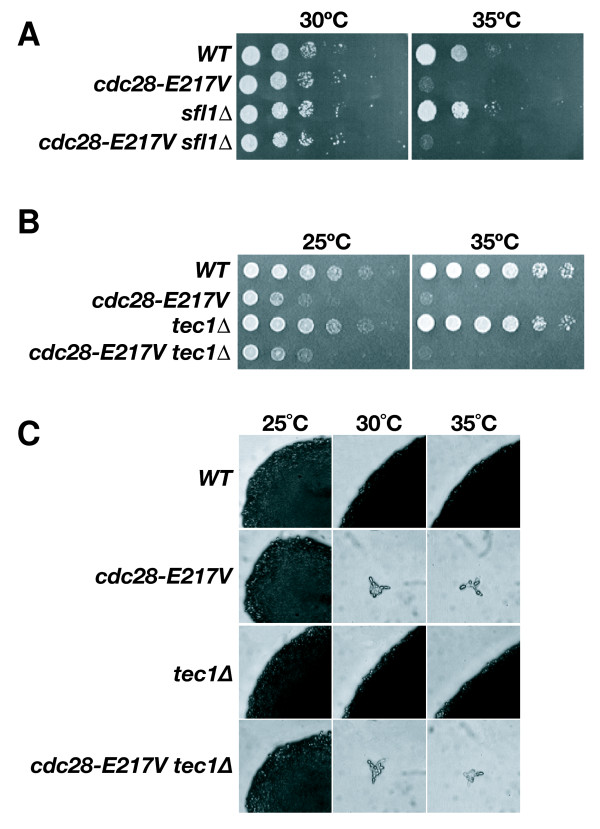
***cdc28-E217V *is not affected by deletion of *TEC1 *or *SFL1***. **A) **Growth of wild type, *sfl1*, *cdc28-E217V *strains and *sfl1 cdc28-E217V *double mutants was examined using of 5-fold serial dilutions of saturated cultures at 30°C and 35°C **B) **Growth of *cdc28-E217V tec1 *double mutants compared to wild-type, *cdc28-E217V *and *tec1 *cells at 25 and 35°C. **C) **Effect of deletion of *TEC1 *on morphology of *cdc28-E217V *at 25, 30 or 35°C.

In filamentous growth signaling, Ras2 activates both the PKA pathway and the Kss1-MAPK cascade [43]. We questioned if *cdc28-E217V *phenotypes were dependent on Ras2 signaling. Deletion of *RAS2 *restored near wild-type growth and morphology to *cdc28-E217V *cells (Fig. 3A,B). Two transcriptional regulators, Flo8 and Sfl1, function as downstream effectors of the PKA pathway [13]. Deletion of *FLO8 *partially alleviated both the growth and morphology defects of *cdc28-E217V *(Fig. 3C,D), whereas deletion of *SFL1 *showed no effect (Fig. 4A). Deletion of *TEC1*, a transcription factor required for MAPK signaling, also did not alter *cdc28-E217V *growth or morphology (Fig. 4B, C). These results suggested that the Ras2-activated PKA pathway specifically impinged on *cdc28-E217V *through Flo8. Providing further confirmation of this connection we found that a high-copy plasmid harboring the cAMP phosphodiesterase *PDE2*, a negative regulator of PKA, suppressed *cdc28-E217V *defects (data not shown) [44,45].

It has also been reported that the PKA pathway, but not the Kss1-MAPK cascade, may be required for activation of filamentation during amino acid starvation by the transcription factor Gcn4 [46]. In accordance, we observed that deletion of *GCN4 *provided some suppression to *cdc28-E217V *cells (Fig. 3E). As a final test we reexamined each of the Cks1-binding mutants for genetic interaction with *FLO8*. Interestingly less that half of these mutants were suppressed by deletion of *FLO8 *(Table 1). These observations indicate that the interaction of PKA through Flo8 with Cdc28 may be highly specific to a particular mechanism of CDK regulation.

### Swe1 is modulated by PKA and Cks1

**Figure 5:**
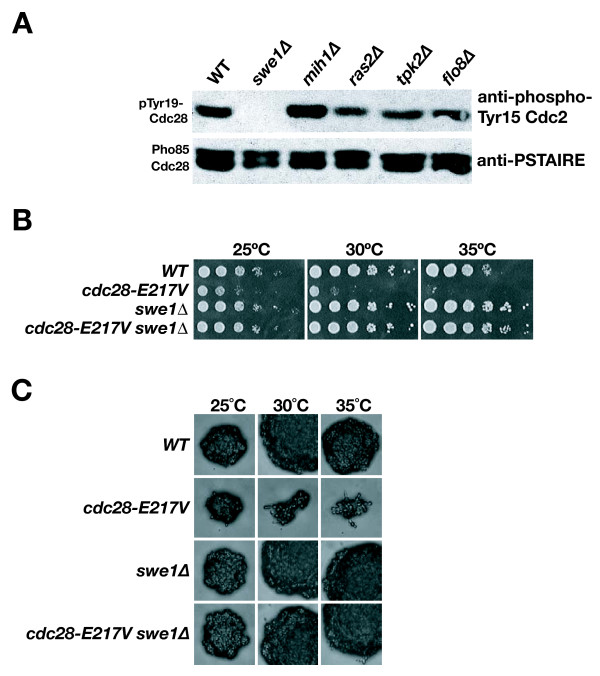
**PKA controls Swe1 regulation of Cdc28**. **A) **Western blot analysis of Cdc28 Tyr19 phosphorylation in *swe1*, *mih1, ras2*, *tpk2*, *flo8*, or wild-type cells grown to log phase at 30°C. **B) **Growth of *cdc28-E217V swe1 *double mutants compared to wild-type, *cdc28-E217V *and *swe1 *cells at 25, 30 or 35°C. **C) **Morphology of *cdc28-E217V swe1 *double mutants at 25, 30 or 35°C compared to wild-type, *cdc28-E217V *and *swe1 *cells.

These results supported the Ras2-activated PKA pathway via Flo8 to be an important regulator of Cdc28. Previous genetic evidence by La Valle and Wittenberg suggested that Swe1 regulation of Cdc28 may be an effector of PKA [18]. Therefore, we directly tested if Swe1 function is modulated by the PKA pathway. We found that deletions of *RAS2*, *FLO8*, or the filamentous-growth specific PKA catalytic subunit *TPK2 *[47] in otherwise wild-type cells each diminished Cdc28 Tyr19 phosphorylation, placing the PKA signaling upstream of Swe1 and Cdc28 (Figure 5A).

Given the relationship between PKA and Swe1, we also tested if *cdc28-E217V *is modulated by *SWE1*. Strikingly, deletion of *SWE1 *conferred suppression of both growth and morphology defects of *cdc28-E217V *(Fig. 5B, C). Again, we asked if these effects were specific to Swe1 or if the absence of any negative regulator of CDK function would improve *cdc28-E217V *defects. Toward examining this question, we observed that deletion of the Cdc28 stoichiometic inhibitor *SIC1 *had only subtle effects on growth of *cdc28-E217V *cells [48].

**Figure 6:**
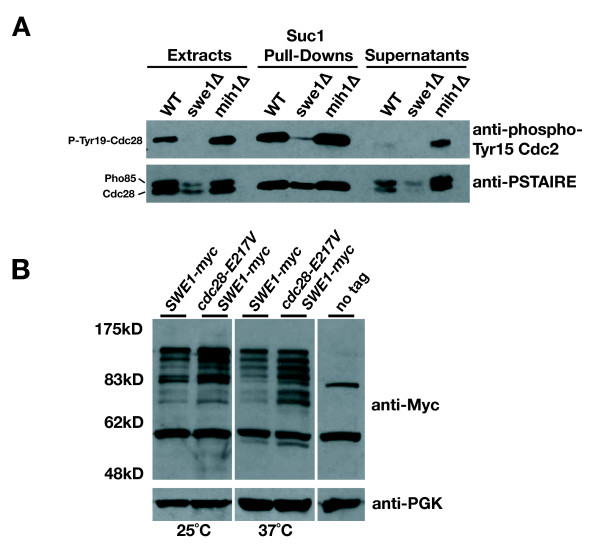
**Increased Swe1 protein levels mediate *cdc28-E217V *defects**. **A) **p13^Suc1 ^beads pull-downs of Tyr19-phosphorylated and unphosphorylated Cdc28 from protein extracts prepared from *swe1 *or *mih1 *deletion strains. **B) **Western blot analysis of Myc-tagged Swe1 protein levels from wild-type and *cdc28-E217V *cells grown to log phase at 25°C and shifted to 37°C, or kept at 25°C for 4 h.

One interpretation of these data is that Swe1 functions upstream of Cks1. Nonetheless, arguing against a role for Swe1 binding or Cdc28 Tyr19 phosphorylation in modulating Cks1 binding, Cdc28 was pulled down by p13^suc1 ^beads to a similar extent from extracts of cells lacking *SWE1 *or its opposing phosphatase, *MIH1 *(Fig. 6A). An alternative explanation is that Cks1 antagonizes Cdc28 binding to or phosphorylation by Swe1. However, GST-Swe1 beads pulled down Cdc28-E217V and wild-type Cdc28 with similar levels, and independent of expression of high copy *CKS1*. In turn, increased Cdc28 Tyr19 phosphorylation was not detected in the mutant cells (data not shown). Together, these results argue against Swe1 and Cks1 binding competitively to Cdc28.

**Figure 7:**
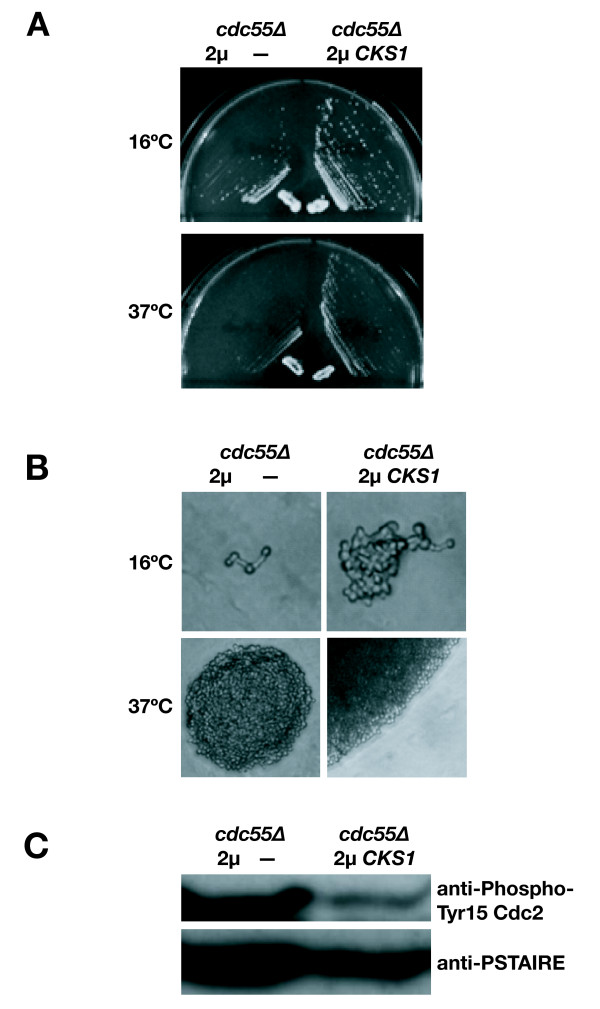
**Cks1 opposes Swe1 function in *cdc55 *mutants**. **A) **Growth and **B) **Morphology of *cdc55 *mutants (W303 background) at 16 and 37°C transformed with high copy *CKS1 *or empty vector. **C) **Cdc28 Tyr19 phosphorylation in *cdc55 *deletion mutants (W303 background) transformed with high copy *CKS1 *or empty vector.

An alternative link to Cks1 is that Swe1 protein might accumulate and/or persist abnormally in *cdc28-E217V *cells. Western blot analysis of the *cdc28-E217V *cells revealed accumulation of Myc epitope-tagged Swe1, which appeared as a range of apparent molecular weights, a pattern previously ascribed to Swe1 phosphorylation [49] (Fig. 6B). To further pursue a role for Cks1 in Swe1 function, we examined cells lacking *CDC55*, a protein phosphatase 2A (PP2A) regulatory subunit that promotes Swe1 degradation. *cdc55 *mutants contain elevated levels of Swe1 and show morphological defects including slow growth, elongated morphology and increased Cdc28 Tyr19 hyperphosphorylation at 16°C, which are relieved by deletion of *SWE1 *[50]. Strikingly, high copy *CKS1 *also strongly suppressed the growth and morphology defects of *cdc55 *mutants and reduced Cdc28 Tyr19 phosphorylation (Fig. 7A, B, C). These results suggest that Cks1 opposes Swe1 accumulation and inhibitory phosphorylation.

## Discussion

Ras2-mediated signaling has been well studied in filamentous growth. Despite this, the extent of crosstalk between the Ras2-MAPK and Ras2-PKA pathways is uncertain. Although traditionally thought of as parallel pathways [8], there has been some evidence that the Kss1 MAPK pathway may inhibit the PKA pathway [51] and that Flo8 and Tec1 participate in shared complexes at promoters [52]. Our results indicate that *cdc28-E217V *cells are modulated by the PKA pathway, most likely via *FLO8 *though the direct targets that mediate this interaction are yet to be established. Deletions of a second downstream component of PKA signaling, *SFL1*, or the Ras2-MAPK transcriptional effector *TEC1 *did not show any genetic interaction with *cdc28-E217V*. La Valle and Wittenberg have shown that deletion of *SWE1 *restores filamentous growth to *flo8 *PKA signaling mutants [18]. Our results extend these findings to suggest the PKA pathway regulates filamentous growth in part by activating Swe1 to phosphorylate Cdc28 Tyr19. Given that *cdc28-E217V *phenotypes partially depend upon the PKA pathway including *FLO8*, but not the MAPK cascade, we suggest that the regulation of Cdc28 by Swe1 and Cks1 is likely specifically mediated by PKA.

A paradigm for signaling cascades that culminate in activation of a CDK regulator has been previously observed in the response of haploid yeast to mating pheromone that galvanizes the Ste-MAPK cascade leading to activation of the MAPK Fus3 [53]. Fus3 positively regulates the CDK inhibitor Far1 directly by phosphorylation and indirectly by transcriptional regulation. In turn, Far1 negatively regulates Cln-associated Cdc28 complexes by inhibiting kinase activity and altering its localization [54-56]. Interestingly, Far1 is also subject to a negative feedback loop involving phosphorylation by Cln-associated Cdc28 that is thought to lead to ubiquitination and degradation of Far1 [57]. We have shown that PKA-signaling pathway via Flo8 regulates Swe1-mediated Cdc28 Tyr19 phosphorylation, and that *SWE1 *mediates *cdc28-E217V *phenotypes. Similarly to Far1, Swe1 is a known Cdc28 substrate [6,22]. Swe1 has been found to make direct contact with the hydrophobic patch of Clb2 initially stabilizing cyclin-Cdc28 complexes prior to its hyperphosphorylation [58], providing a mechanism for feedback inhibition analogous to that of Far1.

*cdc28-E217 *cells exhibit increased levels of Swe1 protein. Complex post-translational modification of Swe1 leads to several different mobility species [49]. Interestingly, each form accumulates in *cdc28-E217V *cells, suggesting that *cdc28-E217V *cells are competent to mediate phosphorylation and ubiquitination of Swe1. This is supported by the observation that Cdc28-E217V exhibits normal kinase activity toward histone H1 and exhibits physical association with Swe1 comparable to wild type Cdc28.

Many CDK substrates are subject to subsequent ubiquitination and proteasome-dependent degradation including cyclins, the stoichiometric inhibitor Sic1, and the securin Pds1 [59,60]. Ubiquitination of proteins is mediated by two complexes, the Skp1-cullin-F-box-protein complex (SCF) and the Anaphase-Promoting Complex (APC). Although SCF complex functions at multiple cell cycle transitions, the APC is thought to function only during mitosis. Substrate specificity of each of these complexes is determined in part by F-box subunits for the SCF, and WD-repeat containing proteins for the APC [26,61]. That the Cdc28-Cks1 physical interaction may be important in the downregulation of Swe1, a CDK substrate, suggests that one role of Cks1 may be to recruit the protein degradation machinery to CDK complexes. Such a role has been reported for the mammalian Cks1, which facilitates ubiquitination and degradation of p27, a CDK stoichiometric inhibitor [62].

In yeast, Cks1 and Cdc28 have been reported to interact with the 26S proteasome [30]. Morris and Reed have proposed a model in which Cks1 recruitment of the proteasome and Cdc28 is important for displacement of CDK from specific promoters in a novel mechanism of transcriptional regulation [29,63]. Crystal studies of mammalian Cks1 have suggested that Cks1 exists as both a monomer and a multimer, although the regulation and function of this transition is not known [64]. Bourne and Tainer have suggested that Cks1 dimerization forms a pocket which may serve to shuttle phospho-proteins [64]. Further recent evidence has shown that Cks1 interacts directly with monoubiquitin and tetraubiquitin [65]. Together, these findings suggest that Cks1 may facilitate transition of ubiquitinated CDK substrates to the proteasome.

Understanding of events leading to Swe1 degradation however has proven elusive. Kaiser and Reed have shown that the SCF subunit Met30 interacts physically with Swe1 and that *met30 *mutants exhibit stabilization of Swe1 protein expressed from an inducible promoter [66]. It is also suggested that *in vitro *translated Swe1 exhibits lower mobility forms on exposure to whole yeast extract that are dependent on the presence of Met30 [66]. However, studies from the Lew laboratory have shown that deletion of *MET30*, unlike *HSL7*, does not enhance the filamentous phenotype caused by deletion of the opposing Tyr19 phosphatase *MIH1 *[22]. Further, they have also shown that double deletion of *MET30 *and another SCF subunit *MET4 *does not stabilize Swe1 protein levels. To further examine Swe1 regulation, McMillan and colleagues measured Cdc28 tyrosine phosphorylation in the absence of each of 13 deletions of non-essential F-box subunits, none of which showed an appreciable increase [22]. Finally, Kellogg has suggested that Swe1 protein is present throughout mitosis and that degradation may not be a primary mechanism of regulation [49,67]. Our findings support that Swe1 protein levels are subject to regulation that depends on Cks1.

That Cks1 may have a role in regulating protein stability may also explain the genetic interaction of *cdc28-E217V *with the PKA pathway. Bolte and Irniger have found that *RAS2-V19 *enhances the growth defects of mutants of the APC through a mechanism that depends on activation of adenylate cyclase [24]. Conversely, deletion of *RAS2 *or overexpression of *PDE2 *diminishes the growth defects of APC mutants. However, it has also been observed that deletion of *RAS2 *may have the opposite effect on SCF mutants, actually enhancing growth defects [25].

## Conclusion

Our results indicate that the RAS-mediated PKA pathway and Cks1 are important regulators of Swe1 and, thereby, of Cdc28-Clb2 mitotic function during yeast filamentous growth. The phenotypes of *cdc28-E217V*, a mutant deficient for proper Cdc28-Cks1 association, are partly dependent on the presence of PKA pathway components and Swe1. Swe1 accumulates in the *cdc28-E217V *cells supporting prior studies showing that association of Swe1 with Clb2 mediates negative feedback provided by CDK phosphorylation [62]. Overexpression of *CKS1 *restores near wild-type growth, morphology and Cdc28 Tyr19 phosphorylation to *cdc55 *mutant cells defective for Swe1 degradation. Future studies elucidating the precise degradation machinery required for Swe1 turnover will be important in understanding the importance of the PKA pathway in regulating Cdc28 CDK activity.

## Methods

### Strain construction

Gene replacement, allele integration, yeast mating, and yeast transformation were as described [3,68]. For genetic analyses, multiple tetratypes were examined. Yeast strains were constructed in the Sigma 1278b background unless indicated. Strains and plasmids were derived from the following:

#### Strains

SKY750 – MATa/α cdc28::TRP1/cdc28::TRP1 his3 [pCT3 CDC28 URA3]; SKY768 – MATa clb1::TRP1 ura3; SKY772 – MATa clb2::LEU2 ura3; SKY901 – MATα swe1::LEU2 ura3; SKY903 – MATα mih1::LEU2 ura3; SKY985 – MATa cln1::URA3 cln2::LEU2 trp1; SKY2244 – MATα ura3 his3 leu2 trp1; SKY2256 – MATa/MATα ura3/ura3 his3/his3 leu2/LEU2 trp1/TRP1; SKY2264 – MATa ura3 his3; SKY2618 – MATa ras2::kanMX6 ura3 his3 leu2; SKY3003 – MATa/MATα cdc28-E217V:kanMX6/cdc28-E217V:kanMX6; SKY3004 – MATa/MATα CDC28/cdc28-E217V:kanMX6; SKY3005 – MATα cdc28-E217V:kanMX6 ura3 his3 leu2; SKY3006 – MATα SWE1-13Myc:kanMX6; SKY3007 – MATa flo8::kanMX6 ura3 his3 trp1; SKY3008 – MATα sfl1::kanMX6 his3; SKY3009 – MATα tec1:kanMX6 ura3 his3; SKY3010 – MATa tpk2::kanMX6 ura3 his3 leu2 trp1; SKY3011 – MATa cdc55::kanMX6 ura3 his3 trp1 (W303); SKY3012 – MATa gcn4::HIS3 ura3 leu2

#### Plasmids

pSKB4250 – YEplac195 *2 μ URA3 GAL*> (gift of M. Solomon); pSKB4251 – YEplac195 *2 μ URA3 GAL>PTC2 *(gift of M. Solomon); pSKB4252 – YEplac195 *2 μ URA3 GAL>PTC3 *(gift of M. Solomon); pSKB4253 – pRS202 *2 μ URA3 CLB2*; pSKB4254 – pRS 202 2 μ *URA3 CAK1*; pSKB4255 – pRS202 2 μ *URA3 CLN1*; pSKB4256 – pRS202 2 μ *URA3 CKS1*; pSKB4257 – pRS202 2 μ *URA3*; pSKB4258 – pCT3 *CEN URA3 CDC28*; pSKB4259 – pGEX-3x-GST-*SWE1 *[69] (gift of J. Thorner); pSKB4260 – pGEX-3x-GST (gift of J. Thorner); pRS413-*cdc28 *library *CEN HIS3*

### Screen for cdc28 mutants

A degenerate-PCR derived library of *cdc28 *mutants cloned into pRS413 was used to transform SKY750 [3]. The transformants were then replica-plated onto 5-Fluoroorotic acid (5-FOA)-containing media to evict the *CEN URA3 CDC28 *plasmid. About 50,000 5-FOA^R ^colonies were plated on synthetic low ammonia dextrose (SLAD) media. Five hundred colonies exhibiting enhanced filamentous growth were isolated and from these, 64 showed dramatically reduced growth at 35°C. Plasmids were rescued and re-introduced in SKY750. After 5-FOA treatment, 39 were found to confer a strong phenotype and were sequenced. Genomic integration of the E217V mutation into SKY2256 was as described [68]. Tetrad analysis and DNA sequencing were used to confirm the integration.

### Bioinformatic Analysis

The effect of polymorphism at E217 was studied as described [38]. Molecular modeling was performed as described [3] using Swiss-Model and Swiss-PDB-Viewer [70,71].

### Spot Tests

Yeast cultures were grown to saturation in rich or selective liquid media at 22°C. Saturated cultures were diluted serially at a ratio of 1/5 and spotted onto rich or selective agar medium. We have found the cdc28 mutants, possibly in part of because of the essential nature of many Cdc28 functions, have a tendency to acquire genomic and intragenic suppressor mutations. Because of this, we repeated spot testing at least four times for each experiment and a representative assay is shown. Each of the experimental groups shown within a given experiment were performed together on the same plate at each temperature. Images were acquired with a Pixera Professional camera on a Zeiss inverted microscope using brightfield illumination and contrast-enhanced in Adobe Photoshop. Magnification for detail of growth and morphology was performed in accord with previously published assessments of filamentation [72,73].

### Biochemical Analyses

Yeast extracts were prepared from log phase cultures by bead beating in buffer containing 50 mM Tris pH 7.6, 10% glycerol, 0.5% NP-40, 2 mM EDTA, 250 mM NaCl, 1 mM phenylmethylsulphonyl fluoride, and protease inhibitor cocktail (Roche). Protein content was determined by Bradford assay. For Western blot analysis, proteins separated by SDS-PAGE were transferred to nitrocellulose membranes, and blocked in TBS-Tween-20 containing 5% milk, or 5% BSA for anti-phospho-Tyr15 Cdc2 antibody (Cell Signaling Technologies). Membranes were probed with polyclonal anti-Myc (A14, Santa Cruz) at 1/1000 dilution, anti-PSTAIRE (Santa Cruz) at 1/500, anti-phospho-Tyr15 Cdc2 (Cell Signaling Technologies) at 1/1000, or anti-PGK (Molecular Probes) at 1/2500. Membranes were washed 3× with TBS-Tween-20 and probed with HRP-linked anti-rabbit or anti-mouse IgG (Amersham) at 1/2500. For p13^Suc1 ^pull-down reactions, 1 mg of protein extract was incubated overnight at 4°C with 50 μ l of p13^Suc1 ^Sepharose bead slurry (Upstate). Beads were washed five times with extract buffer, boiled in sample buffer and the supernatants subjected to SDS-PAGE. For binding assays using GST-Swe1 coated glutathione beads, 1 mg of yeast extract was incubated overnight with 100 μ l of 50% glutathione bead slurry and treated as for p13^Suc1 ^Sepharose beads. GST-Swe1 was isolated from *E. coli *BL21(DE3) plysS harboring pGEX-GST-*SWE1 *[69], a kind gift from Jeremy Thorner. Bacteria were grown to an O.D._600 _of 0.6, induced for 3 h at 37°C with 0.2 mM IPTG, and briefly sonicated in PBS buffer containing protease inhibitor cocktail, 1 mM PMSF, 1 mM Orthovanadate, 0.5 mM DTT, and 1% Triton X-100. Glutathione beads were incubated with 1–2 mg of protein extract overnight at 4°C. GST-Swe1 coated beads were washed five times with PBS and incubated with extracts.

## Abbreviations

(CDK): cyclin-dependent kinase; (SCF): Skp1-cullin-F-box-protein complex; (PKA): protein kinase A; (MAPK): mitogen-activated protein kinase; (PP2A): protein phosphatase 2A; (G1): growth phase 1; (S): synthesis phase; (G2): growth phase 2; (M): mitosis.

## Competing interests

The authors declare that they have no competing interests.

## Authors' contributions

BT generated the hypotheses and conclusions presented and conducted the genetic and biochemical experiments as well as wrote the manuscript. AK made significant contributions to the conception, design, hypotheses and interpretation of data of all of the studies presented, provided substantial technical assistance and optimization of genetic and biochemical assays, developed a large proportion of the specific reagents for testing of genetic and biochemical interactions of cell cycle genes, and extensively reviewed and revised the manuscript. JS and RG participated in repeating and confirming experimental results, as well as compilation and organization of the data. BB performed the primary screen for temperature sensitive *cdc28 *alleles. JC provided intellectual contributions, technical support and aided in review and revision of the manuscript. DC conducted bioinformatic analyses of each of the *cdc28 *alleles. SK conceived the initial study, directed and coordinated all studies, and provided resources and materials for the studies presented.
